# Measures of excess body weight and anthropometry among adult Albertans: cross-sectional results from Alberta’s tomorrow project cohort

**DOI:** 10.1186/s12889-017-4887-2

**Published:** 2017-11-25

**Authors:** Darren R. Brenner, Abbey E. Poirier, Tiffany R. Haig, Alianu Akawung, Christine M. Friedenreich, Paula J. Robson

**Affiliations:** 1 Department of Cancer Epidemiology and Prevention Research, CancerControl Alberta, Alberta Health Services Holy Cross Centre, Room 514, Box ACB, 2210 2nd Street SW, Calgary, AB T2S 3C3 Canada; 2 Department of Oncology and Department of Community Health Sciences, Cumming School of Medicine, University of Calgary, Calgary, Canada; 3 Cancer Measurement, Outcomes, Research and Evaluation (C-MORE), CancerControl Alberta, Alberta Health Services, 10123-99 Street NW, Edmonton, AB T5J 3H1 Canada; 4 Department of Agricultural, Food and Nutritional Science, Faculty of Agricultural, Life and Environmental Sciences, University of Alberta, Edmonton, Canada

**Keywords:** Overweight, Obesity, Cohort, Abdominal adiposity, Chronic disease

## Abstract

**Background:**

Excess body weight during adulthood has been consistently associated with all-cause mortality, cardiovascular disease, and cancer at multiple sites among other chronic diseases. We describe the prevalence of excess body weight and abdominal obesity reported by participants enrolled in Alberta’s Tomorrow Project (ATP).

**Methods:**

ATP is a geographically-based cohort study conducted among adults aged 35–69 years from across the province of Alberta. Participants completed anthropometric measures and health and lifestyle questionnaires at enrolment. Overweight and obese were categorized as a body mass index (BMI) of 25.0–29.9 kg/m^2^ and ≥30 kg/m^2^, respectively. Abdominal obesity was categorized using cut-offs of waist circumference of >94 cm for men and >80 cm for women and waist-tp-hip ratio cut-offs of >0.90 for men and >0.85 for women.

**Results:**

BMI and hip and waist circumference data were obtained from 12,062 men and 18,853 women enrolled between 2001 and 2009. Overall, 76.8% of men and 59.5% of women reported a BMI ≥25 kg/m^2^. The proportions of overweight and obese were significantly higher in older age groups (*p* < 0.001). In addition, the proportion of participants reporting being overweight and obese was higher among lower education (*p* < 0.001) and lower income groups (*p* < 0.001). Overall, approximately two thirds of men and women in ATP cohort reported abdominal obesity. Overweight, obesity and abdominal obesity were all associated with a history of several cardiometabolic chronic conditions including hypertension, heart attack, angina, high cholesterol, stroke and diabetes.

**Conclusion:**

A large majority of ATP participants were overweight and carried excess abdominal fat. Strategies to improve energy balance among Albertans are encouraged and may have a notable impact on future chronic disease burden.

**Electronic supplementary material:**

The online version of this article (10.1186/s12889-017-4887-2) contains supplementary material, which is available to authorized users.

## Background

Excess body weight has been consistently related to a broad array of adverse health outcomes including diabetes, cardiovascular disease and multiple cancer sites, among other chronic diseases and all-cause mortality [1, 2]. The increased caloric density in Western diets, increases in sedentary behaviours/sedentary time and decreases in recreational and transport-related physical activity have all led to a shift in caloric balance at the population level [3]. Sustained imbalance has led to rising obesity rates and specifically, abdominal obesity. Globally, the prevalence of overweight rose from 24.6% in 1980 to 34.4% in 2008 while the prevalence of obesity rose from 6.4% to 12.0%. [4] National surveys conducted by the Public Health Agency of Canada have shown that the prevalence of obesity (body mass index [BMI] >30 kg/m2) is on the rise. Based on a 2011 report, the overall proportion of Canadian adults affected by obesity (>18 years of age) almost doubled between 1978 (13.8%) and 2008 (25.4%) [5].

Two separate analyses using comparable methods estimated that obesity costs the Canadian economy between five and seven billion dollars annually through direct healthcare costs and indirect costs [5, 6]. Therefore, the rising rates of obesity in the population are cause for immediate concern from both public health and economic perspectives.

In this study, we examined the predictors of overweight, obesity and abdominal obesity in the adult Albertan population, which have not yet been well characterized. To quantify the population-level burden of obesity among adult Alberta’s Tomorrow Project (ATP) participants, we estimated the prevalence of overweight/obesity using BMI and waist-to-hip ratio as well as abdominal obesity using waist circumference as reported by participants enrolled in ATP. We also examined the relation between body size and the prevalence of chronic disease in the ATP population.

## Methods

ATP is a geographically-based prospective cohort comprised of 54,942 Albertans between the ages of 35–69 years established to examine the association between various lifestyle factors and chronic disease outcomes. The present analyses were conducted on a subset of the cohort, which included only the 30,915 participants recruited during the first phase of recruitment (2001–2009) who completed the anthropometric section of the Health and Lifestyle Questionnaire (HLQ) at enrolment. Detailed information on the feasibility of recruitment methods, enrollment and data collection for ATP has been previously published [7, 8].

Participants were asked to use a scale to measure weight (in pounds) and were supplied with a tape measure (divided in 1/8″ sections) and detailed instructions, supported by diagrams, to record height (in feet and inches), and waist and hip circumferences (in inches). In addition, Participants were asked to record their anthropometric measurements in a single session at least two hours after a meal, preferably with the assistance of another adult, and to record each measure twice. Participants who were more than 12 weeks pregnant, or less than six months postpartum, were not included in these analyses. Missing, contradictory, ambiguous or out of range anthropometric measures were identified by two data reviewers, and participants were contacted by phone for clarification.

BMI was calculated using participants’ self-reported height and weight measurements (kg/m^2^). BMI categories were classified as normal weight (BMI < 25), overweight (BMI ≥25, <30) and obese (BMI ≥30). Waist and hip circumferences measured in inches were converted to centimetres. Participants were categorized according to World Health Organization (WHO) waist circumference guidelines that recommend using cut-offs of >94 cm for men and >80 cm for women to categorize Western populations into those with abdominal obesity [9]. Additionally, the WHO has provided another set of waist circumference cut-offs that have been found to be associated with substantially higher risk of cardiometabolic complications (>102 cm for men and >88 cm for women). Waist-to-hip ratio was also calculated, and participants were classified according to WHO guidelines that suggest individuals with a waist-to-hip ratio > 0.90 for men and >0.85 for women are at an increased risk for metabolic complications [9].

### Statistical analysis

Descriptive statistics were used to characterize the cohort according to anthropometric measures. Means and standard deviations (SD) summarized continuous variables and frequencies and percentages were used to capture categorical variables. Variable distributions were examined and any outlying or aberrant values were verified using the original questionnaires. Pearson’s chi-square tests were conducted to compare demographic variables and chronic disease outcomes between groups of participants categorized by BMI, waist circumference and waist-to-hip ratio. Logistic regression models for BMI, waist circumference and waist-to-hip ratio were run separately for men and women and were adjusted for age, educational attainment, marital status, annual household income, employment status, smoking status and geographic location (urban/rural). For each anthropometric outcome, linear regression was used to determine if correlations between sociodemographic variables were significantly impacting the associations between the individual sociodemographic variables and BMI and waist circumference. All statistical tests were performed at a 5% level of significance using SAS version 9.2 (SAS Institute, Cary, NC).

## Results

### Body mass index

Table 1 presents the BMI categories by socio-demographic variables. Of the participants enrolled between 2001 and 2009, information on BMI was available from 12,062 men and 18,853 women. Overall, 48.4% (*n* = 5843) of men and 33.2% (*n* = 6251) of women were classified as overweight while 28.4% (*n* = 3424) of men and 26.4% (*n* = 4967) of women were considered obese. Obesity was more prevalent in those who had completed lower levels of education and those were retired, unemployed or students. The prevalence of obesity was lower in higher household income categories (*p* < 0.001). In both men and women, highest rates of obesity were observed in former smokers compared to other smoking statuses.

**Table 1:** Body mass index^a^ of Alberta’s Tomorrow Project participants by socio-demographic categories^b^

		Men (*n* = 12,062) (%)				Women (*n* = 18,853) (%)				
Variables	n	Underweight (0.2)	Normal (23.0)	Overweight (48.4)	Obese (28.4)	n	Underweight (1.1)	Normal (39.4)	Overweight (33.2)	Obese (26.4)
Age										
35–44	3860	0.2	27.1	48.0	24.7	6173	1.5	48.0	29.2	21.3
45–54	4283	0.1	22.5	48.6	28.8	6623	1.0	39.3	33.3	26.5
55–64	2924	0.1	20.0	48.2	31.7	4479	0.7	31.4	36.6	31.3
≥ 65	995	0.2	18.6	50.2	31.1	1578	1.1	29.2	38.2	31.5
missing	0					0				
Education										
High school not completed	1323	0.2	18.5	47.2	34.2	1694	1.1	28.5	34.5	35.9
High school completed	1793	0.3	18.5	49.2	32.1	3916	1.1	35.1	33.8	30.0
Some post-secondary^c^	2248	0.1	20.6	46.9	32.4	4197	1.2	36.4	34.2	28.2
Post-secondary completed	6696	0.2	25.9	49.0	24.9	9042	1.0	44.7	32.2	22.1
missing	2					4				
Marital Status										
Married/Living with partner	9959	0.1	21.8	49.5	28.6	14,251	1.0	40.3	33.6	25.1
Single (never married)	792	0.6	34.2	37.8	27.4	1025	1.6	35.1	28.1	35.2
Divorced/separated/widowed	1308	0.1	25.8	46.6	27.5	3571	1.3	37.2	32.9	28.7
missing	3					6				
Household Income^d^										
< $30,000	1107	0.5	26.8	43.4	29.3	2972	1.6	31.8	31.2	35.4
$30,000–$49,000	2976	0.2	24.9	45.9	29.0	5347	1.0	35.1	34.9	29.0
$50,000–$79,000	3333	0.1	23.2	48.7	28.0	4463	0.9	39.8	32.9	26.4
≥ $80,000	4457	0.1	20.7	51.1	28.1	5507	1.0	47.8	32.1	19.1
missing	189					564				
Occupational Status										
Full Time	9178	0.1	23.5	49.0	27.4	8452	1.0	39.9	32.7	26.4
Part Time/Homemaker	777	0.1	25.2	50.5	24.2	6767	1.2	43.1	32.3	23.4
Unemployed/Student	302	0.0	25.8	42.1	32.1	490	2.0	38.8	32.5	26.7
Retired	1419	0.2	18.3	47.8	33.8	2471	0.8	30.7	37.6	31.0
Other	383	0.5	21.9	39.2	38.4	664	1.4	28.0	31.6	39.0
missing	3					9				
Geographic Location^e^										
Rural	2755	0.1	20.1	48.1	31.7	4563	0.9	34.0	33.8	31.3
Urban	9307	0.2	23.9	48.5	27.4	14,290	1.1	41.2	33.0	24.8
missing	0					0				
Smoking Status										
Current daily smoker	1918	0.4	31.0	45.7	22.9	2862	2.9	40.1	33.4	23.6
Current occasional smoker	420	0.0	21.4	49.5	29.1	544	1.7	41.2	35.9	21.3
Former smoker	4730	0.1	17.0	49.5	33.4	6773	0.6	35.1	33.9	30.4
Never smoker	4985	0.2	25.8	48.4	25.6	8655	0.8	42.5	32.3	24.4
missing	9					19				

Note: all the variables tested were highly significantly different, assessed by Pearson chi-square test (*p* < 0.001)

^a^BMI derived from participant self-reported height and weight. BMI is missing for 157 participants (54 men and 103 women)

^b^Alberta’s Tomorrow Project data at enrolment

^c^Some post-secondary includes combined responses to: some technical school/ college, completed technical school/ college, part of university degree completed

^d^Income data are in response to a question about total household income before tax.

^e^Geographic location determined using postal codes, with rural areas identified according to rural postal code classification (second digit = 0)

In order to compare measures of BMI to other population-based surveys in Canada, we examined the agreement with the Canadian Community Health Survey (CCHS) data collected in 2005 (Cycle 3.1), available through Statistics Canada [10]. Although we restricted the CCHS data to Albertan respondents aged 35–69 years to coincide with the ATP inclusion criteria, the CCHS data did include significantly more participants between the ages of 65–69 (Additional file 1: Table S1). In the ATP cohort, 76.8% of men and 59.6% of women were classified as overweight or obese compared to 65.4% of men and 48.1% of women in the CCHS subsample (Additional file 1: Table S1).

The results of the multinomial logistic regression for overweight, obesity and sociodemographic variables are presented in Table 2. In women, compared to those with a household income ≥$80,000, those with a household income of <$30,000 had a higher odds of being overweight (OR: 1.25, 95% CI: 1.10–1.42) or obese (OR: 2.15, 95% CI: 1.88–2.46). However, in men, having a household income <$80,000 was associated with a decreased odds of being overweight or obese. Living in a rural area compared to living in an urban area was associated with a greater odds of obesity in men (OR: 1.34, 95% CI: 1.18–1.52) and women (OR: 1.37, 95% Ci: 1.25–1.49). Compared to never smokers, male (OR: 1.71, 95% CI: 1.51–1.93) and female (OR: 1.41, 95% CI: 1.30–1.53) former smokers were more likely to be obese.

**Table 2:** Odds of higher body mass index by baseline sociodemographic characteristics in Alberta's Tomorrow Project Cohort from Multinomial Logistic Regression^#^

Sociodemographic factors	Men (*n* = 11,857)†			Women (*n* = 18,259)ǂ		
	≤24.9: Normal^Ұa^	25.0–29.9: Overweight^a^	≥30: Obese^a^	≤24.9: Normal^Ұa^	25.0–29.9: Overweight^a^	≥30: Obese^a^
n(%)	2750 (23.19)	3365 (28.38)	5742 (48.43)	7417 (40.62)	4823 (26.41)	6019 (32.96)
		OR [95% CI]	OR [95% CI]		OR [95% CI]	OR [95% CI]
Age^b^						
35–44	Ref	0.80 [0.63–1.01]	0.81 [0.63–1.05]	Ref	0.54 [0.45–0.64]	0.54 [0.45–0.64]
45–54	Ref	0.90 [0.72–1.13]	1.02 [0.80–1.32]	Ref	0.74 [0.62–0.87]	0.79 [0.66–0.95]
55–64	Ref	0.97 [0.78–1.21]	1.15 [0.91–1.45]	Ref	0.98 [0.83–1.14]	1.06 [0.90–1.24]
65–69*	Ref	Ref	Ref	Ref	Ref	Ref
Education						
High school^c^	Ref	1.05 [0.86–1.29]	0.98 [0.79–1.22]	Ref	0.94 [0.81–1.09]	0.86 [0.74–0.99]
Some post-secondary^d^	Ref	0.89 [0.73–1.08]	0.87 [0.71–1.07]	Ref	0.94 [0.81–1.09]	0.80 [0.69–0.93]
Post secondary completed^e^	Ref	0.71 [0.60–0.84]	0.52 [0.43–0.63]	Ref	0.79 [0.69–0.91]	0.59 [0.51–0.68]
< High school*^f^	Ref	Ref	Ref	Ref	Ref	Ref
Marital status						
Single (never married)^g^	Ref	0.61 [0.51–0.73]	0.80 [0.66–0.98]	Ref	0.96 [0.81–1.13]	1.51 [1.28–1.78]
Divorced/separated/widowed^h^	Ref	0.91 [0.78–1.05]	0.91 [0.77–1.08]	Ref	0.86 [0.78–0.96]	0.89 [0.80–0.99]
Married/Living with partner*^i^	Ref	Ref	Ref	Ref	Ref	Ref
Household Income ($CAN)^j^						
< $30,000	Ref	0.64 [0.53–0.78]	0.63 [0.51–0.78]	Ref	1.25 [1.10–1.42]	2.15 [1.88–2.46]
$30,000 - $49,000	Ref	0.71 [0.63–0.81]	0.73 [0.63–0.84]	Ref	1.34 [1.22–1.48]	1.74 [1.56–1.93]
$50,000 - $79,000	Ref	0.81 [0.73–0.92]	0.81 [0.71–0.92]	Ref	1.18 [1.08–1.30]	1.54 [1.38–1.71]
≥ $80,000*	Ref	Ref	Ref	Ref	Ref	Ref
Occupational status						
Part Time	Ref	0.97 [0.79–1.18]	0.78 [0.62–0.98]	Ref	0.85 [0.78–0.93]	0.73 [0.66–0.80]
Retired	Ref	1.14 [0.94–1.39]	1.36 [1.10–1.68]	Ref	0.96 [0.83–1.10]	0.85 [0.73–0.99]
Other^k^	Ref	0.94 [0.76–1.16]	1.41 [1.13–1.76]	Ref	0.93 [0.84–1.02]	0.96 [0.86–1.06]
Full Time*	Ref	Ref	Ref	Ref	Ref	Ref
Geographic Location^l^						
Rural	Ref	1.19 [1.06–1.34]	1.34 [1.18–1.52]	Ref	1.16 [1.06–1.26]	1.37 [1.25–1.49]
Urban*	Ref	Ref	Ref	Ref	Ref	Ref
Smoking Status						
Current daily smoker	Ref	0.76 [0.66–0.86]	0.63 [0.54–0.73]	Ref	0.98 [0.88–1.09]	0.79 [0.71–0.89]
Current occasional smoker	Ref	1.24 [0.96–1.62]	1.32 [0.99–1.76]	Ref	1.25 [1.02–1.52]	0.95 [0.75–1.21]
Former smoker	Ref	1.45 [1.30–1.61]	1.71 [1.51–1.93]	Ref	1.22 [1.13–1.32]	1.41 [1.30–1.53]
Never smoker*	Ref	Ref	Ref	Ref	Ref	Ref

#- Multinomial logistic regression is modelling the probablity of overweight and obesity

Ұ- Considered the reference category in the multinomial logistic regression

*- Considered reference category for the sociodemographic variable

a- Alberta’s Tomorrow Project baseline data, respondents to height and weight measurements

b- Continuous variable in years placed into categories

c- Completed high school

d- Completed some technical school/college training, or completed technical school/college training, or completed some part of university degree

e- Completed university degree, or completed some part of post-graduate university degree, or completed university post-graduate degree

f- Did not completed grade 8, or completed grade 8 but not high school

g- Single and never have been married

h- Divorced, or separated, or widowed

i- Married, or not married but living with a partner

j- Income data are in response to a question about total household income before tax

k- “Other” includes homemaker, unemployed, student and other category

l- Geographic Location was determined using postal codes, where the “0” as the middle numerical number indicates rural residence

† - Missing in body mass index or in at least one sociodemographic factor and hence excluded from the analysis (*n* = 259)

ǂ- Missing in body mass index or in at least one sociodemographic factor and hence excluded from the analysis (*n* = 697)

In both men and women, the prevalence of overweight and obesity in the ATP cohort was significantly higher in those with a history of hypertension, heart attack, angina, high cholesterol, stroke, diabetes and polyps in the colon (Table 3). In addition, 48.4% of men with Crohn’s disease were overweight.

**Table 3:** Cross-sectional associations between body mass index^a^ category and history of chronic diseases in Alberta’s Tomorrow Project Cohort

Chronic Disease^b^	Men (*n* = 12,062) (%)				Women (*n* = 18,853) (%)			
	n	Underweight and Normal^c^	Overweight	Obese	n	Underweight and Normal^c^	Overweight	Obese
Hypertension	2980	12.4	43.2	44.4	4083	19.3	32.7	48.0
Angina	539	16.1	42.7	41.2	373	22.8	33.5	43.7
High Cholesterol	3787	15.9	49.0	35.2	4562	27.3	37.4	35.2
Heart Attack	353	19.8	40.2	39.9	153	26.2	32.0	41.8
Stroke	109	21.1	47.7	31.2	156	22.4	41.0	36.5
Diabetes	694	13.1	34.7	52.2	814	13.3	22.1	64.6
Polyps in colon	624	19.1	46.0	34.9	891	30.8	35.6	33.7
Crohn’s Disease	62	35.5	48.4	16.1	139	45.3	33.8	20.9

Note: all the variables tested were highly significantly different, assessed by Pearson chi-square test (*p* < .0001)

^a^BMI is derived from participant self-reported height and weight

^b^Chronic disease defined as self-report of a physician diagnosis

^c^Underweight and normal BMI categories grouped together due to small cell sizes

### Waist circumference

Table 4 presents sex-specific waist circumference cut-off categories by demographic variables. Information on waist circumference was available for 12,042 men and 18,787 women. Among participants, 67.4% of men reported waist circumference > 94 cm and 67.0% of women >80 cm. The prevalence of participants with a waist circumference that exceeded the healthy cut-off was higher among older age groups. Conversely, the prevalence of participants meeting healthy waist circumference cut-offs was greater among individuals with higher household income and education attainment (Table 4). Among cohort participants, more women (44.3%) than men (40.7%) were observed in the extremely high risk waist circumference category. In current occasional smokers, 72.9% of participants had a waist circumference in the elevated risk category. For all other smoking statuses, the percent of participants in the elevated risk category ranged from 63.4% to 66.6%.

**Table 4:** Waist circumference and waist-to-hip ratio^a^ categories of Alberta Tomorrow Project participants^b^ by socio-demographic variables

Variable	Waist circumference (%)						Waist-to-hip Ratio (%)		
	Elevated Risk^c^			Extremely High Risk^c^					
	n	No	Yes	n	No	Yes	n	<0.90 (Men), < 0.85 (Women)	≥0.90 (Men), ≥ 0.85 (Women)
Sex									
Men	12,042	32.6	67.4	12,042	59.3	40.7	11,919	14.6	85.4
Women	18,787	33.0	67.0	18,787	55.8	44.3	18,730	61.1	38.9
Age									
35–44	10,019	42.6	57.4	10,019	66.2	33.8	9962	52.1	47.9
45–54	10,871	32.3	67.7	10,871	57.4	42.6	10,805	42.4	57.6
55–64	7375	24.4	75.6	7375	48.4	51.6	7339	35.4	64.7
≥ 65	2564	21.3	78.7	2564	45.7	54.3	2543	31.9	68.1
Education									
High school not completed	2981	23.2	76.8	2981	45.6	54.4	2946	30.1	69.9
High school completed	5685	28.9	71.1	5685	52.7	47.3	5655	43.0	57.0
Some post-secondary^d^	6432	30.4	69.6	6432	53.4	46.6	6391	42.7	57.3
Post-secondary completed	15,725	37.1	62.9	15,725	62.5	37.6	15,652	45.5	54.5
missing	6			6			5		
Marital Status									
Married/Living with partner	24,159	32.8	67.2	24,159	57.9	42.1	24,015	42.7	57.3
Single (never married)	1815	35.8	64.2	1815	55.4	44.6	1803	40.1	59.9
Divorced/separated/widowed	4847	31.8	68.2	4847	53.8	46.2	4823	45.7	54.3
missing	8			8			8		
Household Income^e^									
< $30,000	4050	27.0	73.0	4050	48.1	51.9	4013	39.8	60.2
$30,000 - $49,000	8309	30.1	69.9	8309	53.5	46.5	8259	42.0	58.0
$50,000 - $79,000	7777	32.9	67.1	7777	58.0	42.0	7727	41.7	58.3
≥ $80,000	9955	37.8	62.2	9955	63.2	36.8	9918	45.7	54.3
missing	738			738			732		
Occupational Status									
Full Time	17,586	34.4	65.6	17,586	59.3	40.7	17,473	38.5	61.5
Part Time/Homemaker	7523	35.9	64.1	7523	59.7	40.3	7495	58.5	41.5
Unemployed/Student	795	34.5	65.5	795	55.5	44.5	790	46.1	53.9
Retired	3870	21.9	78.1	3870	46.1	53.9	3845	35.2	64.8
Other	1043	23.7	76.3	1043	44.7	55.3	1034	34.1	65.9
missing	12			12			12		
Geographic Location^f^									
Rural	7286	28.6	71.4	7286	52.6	47.5	7236	40.5	59.6
Urban	23,543	34.2	65.8	23,543	58.6	41.5	23,413	43.8	56.2
Smoking Status									
Current daily smoker	4753	36.6	63.4	4753	58.5	41.5	4719	38.6	61.4
Current occasional smoker	963	27.1	72.9	963	59.2	40.8	953	42.5	57.5
Former smoker	11,470	33.4	66.6	11,470	51.5	48.5	11,399	38.7	61.4
Never smoker	13,612	35.9	64.2	13,612	61.3	38.7	13,547	48.2	51.8
missing	31			31			31		

Note: all the variables tested were highly significantly different, assessed by Pearson chi-square test (*p* < 0.005)

^a^Waist circumference and waist-to-hip ratio derived from participant self-reported measurements. Waist circumference is missing for 243 participants and waist-to-hip ratio is missing for 423 participants

^b^Alberta’s Tomorrow Project data at enrolment. Percentages are reported as frequency of each category divided by total number of participants within rows of sociodemographic factors and separately for men and women, such that proportion of participants falling into each category represent the proportion within that sociodemographic factor

^**c**^Elevated risk for waist circumference defined as >94 cm for men and >80 cm for women. Extremely high risk defined as >102 cm for men and >88 cm for women

^d^Some post-secondary includes combined responses to: some technical school/ college, completed technical school/ college, some part of university degree completed

^e^Income data are in response to a question about total household income before tax

^f^Geographic location determined using postal codes, with rural areas identified according to rural postal code classification (second digit = 0)

Table 5 presents the results of the logistic regression model for waist circumference and sociodemographic variables. Compared to men who work full-time, men who are retired have a higher odds of having a waist circumference > 94 cm (OR: 1.29, 95% CI: 1.08–1.53) and >102 (OR: 1.27, 95% CI: 1.09–1.47). The odds of having a waist circumference in the elevated risk category was greater for both rural men and women (OR: 1.19, 95% CI: 1.08–1.32) and women (OR: 1.19, 95% CI: 1.10–1.29). Compared to never smokers, former smokers were more likely to have a waist circumference in the elevated risk category in men (OR: 1.48, 95% CI: 1.35–1.62) and women (OR: 1.35, 95% CI: 1.26–1.45).

**Table 5:** Odds of higher waist circumference and waist-to-hip ratio by baseline sociodemographic characteristics in Alberta's Tomorrow Project Cohort from binary logistic regression^#^

Sociodemographic Factors	Waist circumference^a^ (*n* = 11,807)†						Waist-Hip Ratio^a^ (*n* = 11,857)ǂ		
	Elevated Risk			Extremely High Risk					
	≤94 cm (M), ≤80 cm (W)*	> 94 cm (men)	>80 (women)	≤102 cm (M), ≤88 (W)*	>102 cm (men)	>88 (women)	<0.90 (M), <0.85 (W)*	≥0.90 (men)	≥0.85 (women)
	OR [95% CI]								
Age^b^									
35–44	Ref	0.54 [0.44–0.66]	0.44 [0.38–0.53]	Ref	0.60 [0.50–0.72]	0.54 [0.46–0.62]	Ref	0.39 [0.29–0.51]	0.45 [0.39–0.52]
45–54	Ref	0.80 [0.65–0.98]	0.67 [0.57–0.79]	Ref	0.84 [0.70–0.99]	0.76 [0.66–0.88]	Ref	0.64 [0.48–0.85]	0.68 [0.59–0.78]
55–64	Ref	0.96 [0.79–1.16]	0.96 [0.82–1.13]	Ref	1.05 [0.90–1.24]	1.03 [0.90–1.17]	Ref	0.98 [0.75–1.29]	0.89 [0.79–1.02]
65–69*	Ref	Ref	Ref	Ref	Ref	Ref	Ref	Ref	Ref
Education									
High school^c^	Ref	0.93 [0.78–1.10]	0.88 [0.76–1.01]	Ref	0.89 [0.77–1.03]	0.86 [0.76–0.98]	Ref	0.88 [0.70–1.12]	0.87 [0.77–0.99]
Some post-secondary^d^	Ref	0.87 [0.74–1.02]	0.84 [0.73–0.97]	Ref	0.91 [0.79–1.05]	0.85 [0.75–0.97]	Ref	0.85 [0.68–1.07]	0.85 [0.75–0.96]
Post secondary completed^e^	Ref	0.63 [0.54–0.73]	0.73 [0.64–0.84]	Ref	0.65 [0.57–0.74]	0.67 [0.60–0.75]	Ref	0.63 [0.51–0.77]	0.71 [0.64–0.80]
< High school*^f^	Ref	Ref	Ref	Ref	Ref	Ref	Ref	Ref	Ref
Marital status									
Single (never married)^g^	Ref	0.74 [0.63–0.86]	1.09 [0.94–1.27]	Ref	0.89 [0.76–1.05]	1.38 [1.20–1.58]	Ref	1.00 [0.82–1.23]	1.19 [1.03–1.37]
Divorced/separated/widowed^h^	Ref	0.85 [0.74–0.96]	0.83 [0.76–0.91]	Ref	0.94 [0.83–1.07]	0.93 [0.85–1.02]	Ref	0.93 [0.78–1.10]	0.91 [0.83–0.99]
Married/Living with partner*^i^	Ref	Ref	Ref	Ref	Ref	Ref	Ref	Ref	Ref
Household Income ($CAN)^j^									
< $30,000	Ref	0.82 [0.70–0.97]	1.78 [1.58–2.00]	Ref	0.92 [0.79–1.08]	1.78 [1.60–1.99]	Ref	0.82 [0.66–1.01]	1.68 [1.51–1.88]
$30,000 - $49,000	Ref	0.92 [0.82–1.02]	1.53 [1.40–1.67]	Ref	0.91 [0.82–1.02]	1.56 [1.43–1.69]	Ref	1.06 [0.92–1.22]	1.35 [1.23–1.47]
$50,000 - $79,000	Ref	0.96 [0.87–1.06]	1.36 [1.25–1.48]	Ref	0.93 [0.85–1.03]	1.37 [1.26–1.49]	Ref	1.07 [0.94–1.22]	1.25 [1.15–1.37]
≥ $80,000*	Ref	Ref	Ref	Ref	Ref	Ref	Ref	Ref	Ref
Occupational status									
Part Time	Ref	0.99 [0.83–1.17]	0.80 [0.74–0.87]	Ref	0.89 [0.76–1.05]	0.82 [0.75–0.89]	Ref	1.12 [0.88–1.44]	0.89 [0.82–0.96]
Retired	Ref	1.29 [1.08–1.53]	1.00 [0.87–1.15]	Ref	1.27 [1.09–1.47]	0.96 [0.85–1.08]	Ref	0.95 [0.75–1.20]	0.98 [0.87–1.11]
Other^k^	Ref	1.38 [1.15–1.66]	0.95 [0.87–1.04]	Ref	1.39 [1.17–1.64]	1.00 [0.92–1.09]	Ref	1.10 [0.87–1.38]	1.03 [0.95–1.13]
Full Time*	Ref	Ref	Ref	Ref	Ref	Ref	Ref	Ref	Ref
Geographic Location^l^									
Rural	Ref	1.19 [1.08–1.32]	1.19 [1.10–1.29]	Ref	1.16 [1.06–1.27]	1.17 [1.09–1.25]	Ref	1.24 [1.09–1.41]	1.05 [0.97–1.12]
Urban*	Ref	Ref	Ref	Ref	Ref	Ref	Ref	Ref	Ref
Smoking Status									
Current daily smoker	Ref	0.81 [0.72–0.91]	1.01 [0.91–1.11]	Ref	0.93 [0.83–1.05]	1.03 [0.94–1.13]	Ref	1.14 [0.98–1.33]	1.38 [1.26–1.51]
Current occasional smoker	Ref	1.15 [0.92–1.42]	1.25 [1.03–1.51]	Ref	1.31 [1.06–1.61]	1.03 [0.86–1.24]	Ref	0.97 [0.75–1.26]	1.27 [1.06–1.53]
Former smoker	Ref	1.48 [1.35–1.62]	1.35 [1.26–1.45]	Ref	1.49 [1.36–1.62]	1.29 [1.21–1.38]	Ref	1.52 [1.35–1.72]	1.25 [1.17–1.34]
Never smoker*	Ref	Ref	Ref	Ref	Ref	Ref	Ref	Ref	Ref

#- Binary logistic regression is modelling the probability of higher waist circumference and waist-to-hip ratio categories

Ұ- Considered the reference category in a binary logistic regression

*- Considered reference category for the sociodemographic variable

a- Alberta’s Tomorrow Project baseline data, respondents to waist and hip circumference

b- Continuous variable in years placed into categories

c- Completed high school

d- Completed some technical school/college training, or completed technical school/college training, or completed some part of university degree

e- Completed university degree, or completed some part of post-graduate university degree, or completed university post-graduate degree

f- Did not completed grade 8, or completed grade 8 but not high school

g- Single and never have been married

h- Divorced, or separated, or widowed

i- Married, or not married but living with a partner

j- Income data are in response to a question about total household income before tax etc.

k- “Other” includes homemaker, unemployed, student and other category

l- Geographic Location was determined using postal codes, where the “0” as the middle numerical number indicates rural residence

† − Missing in waist circumference or in at least one sociodemographic factor and hence excluded from the analysis (*n* = 309)

ǂ- Missing in waist-to-hip ratio or in at least one sociodemographic factor and hence excluded from the analysis (*n* = 259)

Significant positive associations were observed between both the elevated risk and extremely high risk waist circumference categories and a history of hypertension, angina, high cholesterol, stroke, diabetes and polyps in the colon (Table 6). In addition to these associations, those in the extremely high risk waist circumference category were also more likely to have a history of Crohn’s disease (Table 6).

**Table 6:** Cross-sectional associations between waist circumference, hip-to-waist ratio^a^ and history of chronic diseases in Alberta’s Tomorrow Project Cohort

Chronic Disease^b^	Waist Circumference (%)						Waist-to-Hip Ratio		
	Elevated Risk^c^			Extremely High Risk^c^					
	n	No	Yes	n	No	Yes	n	<0.90 (Men), < 0.85 (Women)	≥0.90 (Men), ≥ 0.85 (Women)
Hypertension	7029	14.7	85.3	7029	35.0	65.0	6980	26.7	73.3
Angina	907	18.2	81.8	907	38.3	61.7	895	22.5	77.5
High Cholesterol	8323	21.3	78.7	8323	45.2	54.8	8265	29.7	70.3
Heart Attack	504	19.3	80.8	504	39.5	60.5	500	15.2	84.8
Stroke	264	18.9	81.1	264	36.7	63.3	261	27.6	72.4
Diabetes	1495	11.9	88.1	1495	24.5	75.5	1478	16.0	84.0
Polyps in colon	1507	21.5	78.5	1507	45.0	55.0	1501	33.0	67.0
Crohn’s Disease	202	30.7	69.3	202	61.9	38.1	202	42.1	57.9

Note: all the variables tested were highly significantly different, assessed by Pearson chi-square test (*p* < 0.001)

^a^Waist circumference and waist-to-hip ratio derived from participant self-reported measurements. Waist circumference measurements are missing for 243 participants, waist-to-hip ratio missing for 423 participants

^b^Chronic disease defined as self-report of a physician diagnosis

^c^Elevated risk for waist circumference defined as >94 cm for men and >80 cm for women. Extremely high risk defined as >102 cm for men and >88 cm for women

### Waist-to-hip ratio

Sex-specific waist-to-hip ratio cut-off categories by demographic variables are also presented in Table 4. Information on waist-to-hip ratio was available for 11,919 men and 18,730 women in ATP cohort. Overall, 85.4% of men and 38.9% of women had waist-to-hip ratios that exceeded healthy cut-offs of ≥0.90 and ≥0.85, respectively. The prevalence of participants exceeding the cut-offs for waist-to-hip ratio was higher among older age groups, and was lower in groups that reported higher educational attainment and greater household income.

Based on the logistic regression model (Table 5), living in a rural area compared to an urban area was associated with a higher odds of having a waist-to-hip ratio above the cut-off in men (OR: 1.24, 95% CI: 1.09–1.41). In women, having a household income <$30,000 was associated with a higher odds of having a waist-to-hip ratio above the cut-off (OR: 1.68, 95% CI: 1.51–1.88). Compared to never smokers, the odds of having a waist-to-hip ratio above the cut-off was greater for former smokers in men (OR: 1.52. 95%CI: 1.35–1.72) and women (OR: 1.25, 95% CI: 1.17–1.34).

The prevalence of participants with a waist-to-hip ratio above the cut-off was higher among those with a history of hypertension, angina, high cholesterol, stroke, diabetes and Crohn’s disease (Table 6).

## Discussion

In the ATP cohort, 77% of men and 60% of women had a BMI that exceeded the normal range, suggesting that more than two thirds of the participants were overweight or obese. The CCHS reported that 65.4% of men and 48.1% of women were classified as having a BMI above the normal range (Additional file 1: Table S1). Similar to the results observed in the present study, earlier analyses conducted on the first 11,865 participants enrolled in ATP showed a higher proportion of obesity in Alberta Tomorrow Project participants than CCHS (Cycle 1.1) participants residing in Alberta [7]. Some of the difference in prevalence of obesity between the CCHS and ATP could be due to the time periods in which the data were collected. The CCHS data were collected in 2005, but 40.8% of the ATP data were collected between 2006 and 2009, when rates of overweight and obesity were higher.

BMI has been widely criticized as a measure of body size in health research due to its methodological limitations, particularly in terms of failing to address body composition [11]. When examining waist circumference, we observed high rates of abdominal obesity. Over 40% of the cohort reported waist circumferences that are associated with a significantly elevated risk of metabolic complications [9], with a higher proportion observed among women. These sex differences highlight the limitations of BMI, where more men are classified as overweight, potentially because of their higher muscle mass, as compared to using waist circumference categories. In contrast, 57% of participants in the cohort had a waist-to-hip ratio above the cut-off established by the WHO, with substantially higher prevalence observed among men compared to women, which is expected given the differences in male and female body composition.

Consistent with previous research, excess body weight, as measured by BMI, waist circumference and waist-to-hip ratio, was more prevalent among ATP participants with lower household income and education [9]. Across the different measures, the proportion of the study population with excess body weight was slightly higher in rural populations. Similar trends have been observed in other Canadian population-based surveys [12, 13] as well in the United States [14]. Although the reasons for greater prevalence of obesity in participants living in rural areas are not clear and warrant further investigation, one U.S. study showed that those living in rural areas were less physically active than those living in urban areas, which could contribute to the higher obesity rates [15].

Participants in ATP cohort who had excess body weight were more likely to report a history of hypertension, angina, diabetes, polyps in the colon, high cholesterol and Crohn’s disease. In addition, these participants were more likely to have had a heart attack or stroke. Although the biological mechanism linking overweight/obesity and chronic disease depends on the specific outcome, in general it involves bioactive mediators being released from adipose tissues, which in turn can lead to insulin resistance, inflammation and changes in blood pressure, lipid concentrations and coagulation [16].

Several limitations to the data presented in this analysis should be discussed. These data should not be considered representative of the Alberta population as a whole. In addition, misclassification by respondent bias is a potential limitation that should be considered. It has been previously reported that weight-related values are underreported, while height is exaggerated [17]. The cohort does not include young adults (age 18- < 35), who have been shown to have a slightly lower prevalence of obesity than the general Alberta population [13]. We observed several significant associations in this analysis which may be in part due to the large sample sizes being examined.

Although we did not evaluate prospective trends in this analysis, national data suggest that abdominal obesity is especially on the rise [18, 19]. Overweight/obesity in Canada is widely known to be associated with various adverse health outcomes [20]. Furthermore, complications of obesity expand beyond physical health into possible psychological concerns [21] that can have an equally negative effect on the population and the healthcare system. Implementation of existing, and development of novel, interventions targeted at reducing obesity in Alberta should be a public health priority. Including measures of excess body weight in follow-up questionnaires will be important for understanding how measures of BMI, waist circumference and waist-to-hip ratio change over time and what impact these changes have on disease outcomes.

There are a wide range of factors that could be affecting the overweight/obesity trends in the Alberta cohort. It has been demonstrated repeatedly that socioeconomic factors have an overarching impact. For instance, it has been reported that 34% of BMI status can be attributed to educational background, marital status, smoking status and to a less significant extent, sleep deprivation based on results from a cohort study [22]. In the current study, education (*p* < 0.001), marital status (*p* < 0.001) and smoking status (*p* < 0.001) were significantly associated with BMI in both men and women. These relationships between socioeconomic status and BMI are possibly attributable to the differential distribution of certain lifestyle behaviours across socioeconomic categories [23].

## Conclusion

These analyses suggest that excess body weight, as measured by BMI, waist circumference and waist-to-hip ratio is highly prevalent in the cohort. The prevalence of overweight/obesity was higher in the lower education and income groups. Our data further implies that there are multiple chronic conditions associated with excess body weight. Multi-faceted approaches targeting the governmental, community and individual-level changes will be required to improve the energy balance of Albertans and improve subsequent health outcomes.

## Additional file

Additional file 1: Table S1. Age and BMI distributions at enrolment in Alberta’s Tomorrow Project compared with the Canadian Community Health Survey (Cycle 3.1). Table S1 compares the age and BMI distributions of Alberta’s Tomorrow project participants at enrolment and Canadian Community Health Survey respondents (cycle 3.1) from Alberta. (DOCX 17 kb)

## Abbreviations

ATP: Alberta’s Tomorrow project
BMI: Body mass index
CCHS: Canadian Community Health Survey
HLQ: Health and Lifestyle Questionnaire
SD: Standard deviation
VIF: Variance inflation factor
