# Supplementary material for: Measures of excess body weight and anthropometry among adult Albertans: cross-sectional results from Alberta’s tomorrow project cohort
Source: BMC Public Health. 2017 Nov 25;17:899. doi: 10.1186/s12889-017-4887-2 (PMC5702087; doi:10.1186/s12889-017-4887-2)
Supplement: Additional file 1: Table S1. — Age and BMI distributions at enrolment in Alberta’s Tomorrow Project compared with the Canadian Community Health Survey (Cycle 3.1). Table S1 compares the age and BMI distributions of Alberta’s Tomorrow project participants at enrolment and Canadian Community Health Survey respondents (cycle 3.1) from Alberta. (DOCX 17 kb) [file 12889_2017_4887_MOESM1_ESM.docx]

**Additional file 1 Table S1.** Age and BMI distributions at enrolment in Alberta’s Tomorrow Project compared with the Canadian Community Health Survey (Cycle 3.1)

|  |  | | | | | | | |  |
| --- | --- | --- | --- | --- | --- | --- | --- | --- | --- |
| Socio-demographic domains  (self-reported) | | Men | | |  | Women | | |  |
|  |  | HLQ^*^ | CCHS 3.1^†^ |  | | | HLQ^*^ | CCHS 3.1^†^ | |
|  |  | (*n*= 12,116) | (*n*= 3,376) |  | | | (*n*= 18,956) | (*n*= 3,915) | |
|  |  | % | % |  | | | % | % | |
| **Sex** | | 39.0 | 49.8 |  | | | 61.0 | 50.2 | |
| **Age (years)** | |  |  |  | | |  |  | |
| 35-44 | | 32.0 | 32.8 |  | | | 32.8 | 30.9^§^ | |
| 45-54 | | 35.5 | 30.8^§^ |  | | | 35.1 | 29.8^§^ | |
| 55-64 | | 24.2 | 18.6^§^ |  | | | 23.7 | 18.2^§^ | |
| 65-69 | | 8.3 | 17.9^§^ |  | | | 8.3 | 21.1^§^ | |
| Missing (n) | | 0 | 0 |  | | | 0 | 0 | |
| **BMI (kg/m^2^)**^‡^ | |  |  |  | | |  |  | |
| < 18.5 | | 0.2 | 0.4^§^ |  | | | 1.1 | 2.9^§^ | |
| 18.5 - 24.9 | | 23.0 | 34.2^§^ |  | | | 39.4 | 49.0^§^ | |
| 25.0 - 29.9 | | 48.4 | 45.7^§^ |  | | | 33.2 | 31.4^§^ | |
| > 30.0 | | 28.4 | 19.7^§^ |  | | | 26.4 | 16.7^§^ | |
| Missing (n) | | 54 | 34 |  | | | 103 | 199 | |
|  |  | | | | | | | |  |
| ^*^ HLQ, Health and Lifestyle Questionnaire (survey at enrolment) | | | | | | | | |  |
| ^†^ Canadian Community Health Survey 3.1 (2005) Alberta weighted data, restricted to CCHS 3.1 Albertan respondents aged 35–69 years as per Alberta’s Tomorrow Project (ATP) inclusion criteria. | | | | | | | | |  |
| ^‡^BMI - derived from participant self-reported height and weight | | | | | | | | |  |
| ^§^ *p*<.05 (HLQ compared to CCHS3.1) | | | | | | | | |  |
|  | | | | | | | | |  |
